# Development and evaluation of a multi-axis loading simulator for the spine, pelvis, and lumbosacral region

**DOI:** 10.1038/s41598-026-68816-9

**Published:** 2026-08-31

**Authors:** Toni Wendler, Robin Heilmann, Hannah Müller, Jean-Pierre Fischer, Andreas Höch, Christian Kleber, Christoph-E. Heyde, Stefan Schleifenbaum

**Affiliations:** 1 Department of Orthopedic, Trauma and Plastic Surgery, University of Leipzig Medical Center, Liebigstraße 20, D-04103 Leipzig, Germany; 2 ZESBO - Center for Research on Musculoskeletal Systems, Faculty of Medicine, Leipzig University, Semmelweisstr. 14, D-04103 Leipzig, Germany

**Keywords:** Biomechanical loading simulator, Range-of-motion spine, Multiaxial loading, Implant investigation of spine and pelvis, Biomechanical testing, Anatomy, Engineering, Health care, Medical research

## Abstract

The treatment of injuries and disorders of the pelvis, lumbar spine and lumbosacral junction remains challenging due to rising case numbers, complex anatomy and high biomechanical demands. Despite advances in surgical techniques and implant design, problems regarding stability and long-term functionality persist, requiring individualized solutions and a thorough biomechanical assessment. Biomechanical studies are essential for assessing implant performance under physiological conditions. This study therefore aims to develop and validate a versatile biomechanical loading simulator capable of reproducing common test protocols, improving the comparability of results and enabling a systematic evaluation of innovative implant and treatment concepts. A loading simulator with three active degrees of freedom (driven by controlled servo motors) and two passive degrees of freedom (low-friction linear slide bearings) was developed. Five pelvic specimens with an intact lumbar spine up to L2 were first subjected to a range-of-motion (ROM) analysis and subsequently to cyclic dynamic loading to test the functionality of the simulator. The ROM analysis resulted in mean absolute deviations (MAD) of less than 7.50 N for all translational degrees of freedom (DOF) and less than 0.87 Nm for the two unloaded rotational DOF. The maximum moment in the loading direction deviated by a MAD of less than 0.18 Nm. The cyclic loading protocol was achieved in the vertical force direction (60% body weight (BW)) with a MAD of less than 0.76% BW. The maximum and minimum moments to be applied in extension-flexion (± 5 Nm) were realized with a MAD of less than 0.23 Nm. In summary, the simulator developed demonstrates a high degree of accuracy, reproducibility and flexibility. It therefore provides a suitable platform for the biomechanical investigation of implants and stabilization concepts in the pelvic and spinal regions and offers a promising basis for future experimental studies.

## Introduction

 The treatment of disorders and injuries affecting the pelvis, the lumbar spine and the lumbosacral junction remains a major challenge in orthopaedic and trauma surgery. Every year, numerous patients are treated for pelvic fractures^1–3^ and fractures in the lumbar spine and lumbosacral region^2^, with epidemiological surveys showing rising case numbers, for example a 21.7% increase between 2009 and 2019 for the lumbar spine^4^. These regions are critical not only because of their anatomical complexity and high biomechanical stress, but also because they are frequently affected by associated injuries and stress-related complications.

Despite advances in surgical techniques and implant designs, clinical difficulties and unresolved issues persist. Stabilization concepts and long-term functionality of implants, particularly in the pelvic and lumbar regions, remain the subject of intensive research^5,6^. The heterogeneous load situation, inter-individual anatomical variability, and the high demands on both stability and mobility require customized solutions that go beyond conventional implant designs. Given this, there is a high demand for further development of implants and their biomechanical evaluation. Biomechanical studies provide crucial insights into the behavior of implants under physiologically relevant loads and make a significant contribution to optimizing design, materials and implantation strategy.

Internationally standardized mechanical testing methods have become established for the preclinical evaluation of spinal implants, enabling a comparable characterization of implant performance. For example, ASTM F1717 describes the testing of posterior stabilization systems in a vertebrectomy model without ventral support, comprising both static loading and cyclic fatigue testing under combined tension–compression–bending conditions. In a similar manner, the ISO 12,189 standard addresses the fatigue testing of flexible or dynamic spinal implants while taking anterior support into account. Here, sinusoidal compression-bending loads at physiologically relevant force levels are applied. ASTM F2624, by contrast, focuses on investigating implant behavior under load transfer to the adjacent vertebral bodies and serves in particular to assess the risk of subsidence under axial compressive loading. Overall, these standards form the basis for the current assessment of the mechanical safety and performance of spinal implants. However, they focus primarily on simplified load cases and only partially capture the complex loading conditions of the spine in the context of the individual bone-implant interface.

In addition to the testing standards, a wide range of biomechanical loading protocols for the pelvic, lumbar, and lumbosacral regions is described in the scientific literature, covering various loading scenarios, from simple static tests to dynamic multi-axial loads^7–10^.

In their review articles, Costi et al.^11^ and Borrelli et al.^12^ provide a very detailed overview of the variety of methods applied to the lumbar spine, which generally describe a more physiologically realistic load application than the test standards and take a realistic bone-implant interface into account. This diversity highlights the need for a loading simulator that can be flexibly adapted to different testing protocols to ensure a valid and reproducible evaluation.

There are already a multitude of load simulators in biomechanical research. In particular, simulators designed specifically for the spine are frequently used^13–16^. However, most of these simulators are not designed to mount and test pelvises, or pelvises and spines as a unit. Moreover, there are only a few test rigs designed to simulate loads on the pelvis^17^. In contrast, the aim of this study was to develop a versatile biomechanical loading simulator capable of applying multiaxial loading protocols to the spine, the lumbosacral region and the pelvis to provide a system for the systematic evaluation of innovative implant and treatment concepts. Furthermore, the evaluation and demonstration of functionality should be carried out using two testing scenarios applied to human specimens of the lumbosacral region.

## Materials and methods

### Simulator setup

**Fig. 1:**
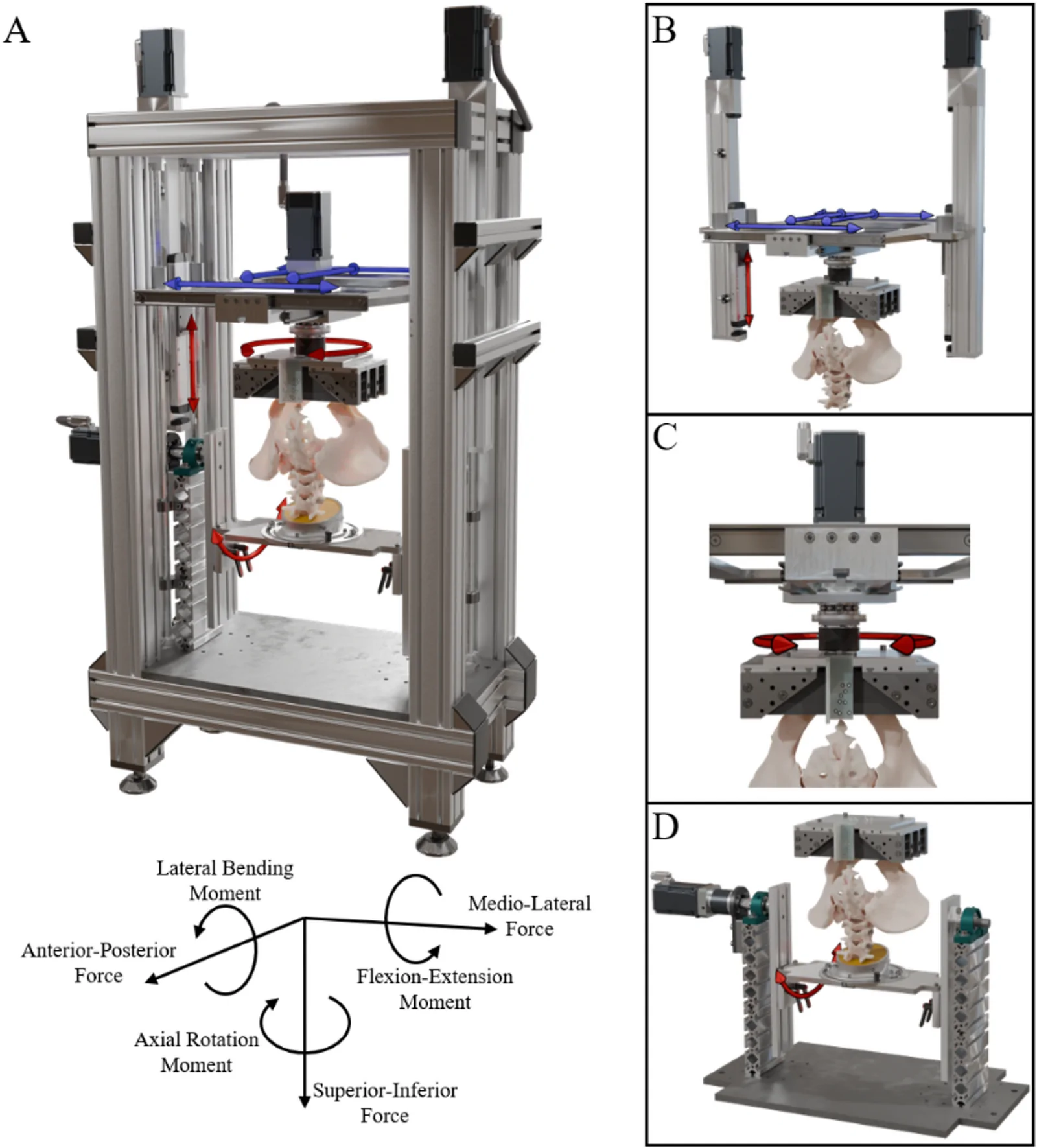
(**A**) Design of a multi-axial loading simulator for spine, pelvis and the lumbosacral system; (**B**) Detailed view of the active crossbeam and passive XY table; (**C**) Detailed view of the active axis for initiating rotation; (**D**) Detailed view of the active axis for initiating flexion and extension; The actively movable degrees of freedom (DOF) are marked with red arrows. The passively movable DOF are marked with blue arrows.

The testing rig for simulating the movement and loading of pelvis and spine essentially consists of a frame, a movable crossbeam, a tilting platform and an XY table with a rotary drive mounted at its center (see Fig. 1). The frame is constructed using aluminium profiles (Profile 8 80 × 40 light, item Industrietechnik, Solingen, Germany). The crossbeam is moved by two servo motors (AM8132, Beckhoff Automation, Verl, Germany) with a connected linear axis (PAS42, Schneider Electric, Rueil-Malmaison, France) and simulates vertical compression. The tilting platform is rotated by a rotary servo motor with a connected gearbox (AM8131 + AG2250 with gear ratio 100, Beckhoff Automation, Verl, Germany) and simulates the extension and flexion movements. The third drive, which is connected to the XY table on the crossbeam via a low-friction ball bearing, also consists of a servo drive with a gearbox (AM8131 + AG2250 with a gear ratio of 12, Beckhoff Automation, Verl, Germany) and simulates the axial rotational movement or moment on the specimen. The XY table is equipped with low-friction linear ball bearings (GN 2402, Otto Ganter, Furtwangen, Germany) and allows for passive movement in the transverse plane to avoid shear forces, as recommended by Wilke^18^. Previous studies have shown that this type of bearing is particularly well suited for preventing shear forces^13,19^.

This configuration enables the simulator to actively apply movement and/or load to the specimen in three degrees of freedom. In two further degrees of freedom, any forces that arise are passively eliminated.

The forces and moments acting on the system are measured using a 6DOF sensor (K6D40, ME-Meßsysteme, Hennigsdorf, Germany), which is mounted below the XY table on the shaft of the rotary drive responsible for torsion. Axis control and sensor signal processing were implemented using an automation system from Beckhoff Automation customized to the simulator, which is essentially based on an industrial PC and I/O components connected via EtherCAT. The simulator software was developed using TwinCAT 3.1 in the TwinCAT XAE Shell and handles the conversion and evaluation of the sensor signals, as well as the control and operation of the servo drives and other peripheral devices.

As many biomechanical studies require an external optical measurement system to record, for example, fracture gap movements or multisegmental movements of the spine, the simulator is equipped with a trigger output that can be used to control external measuring devices. However, this feature was not used in the present study.

### Motion and load capabilities & control algorithm

**Fig. 2:**
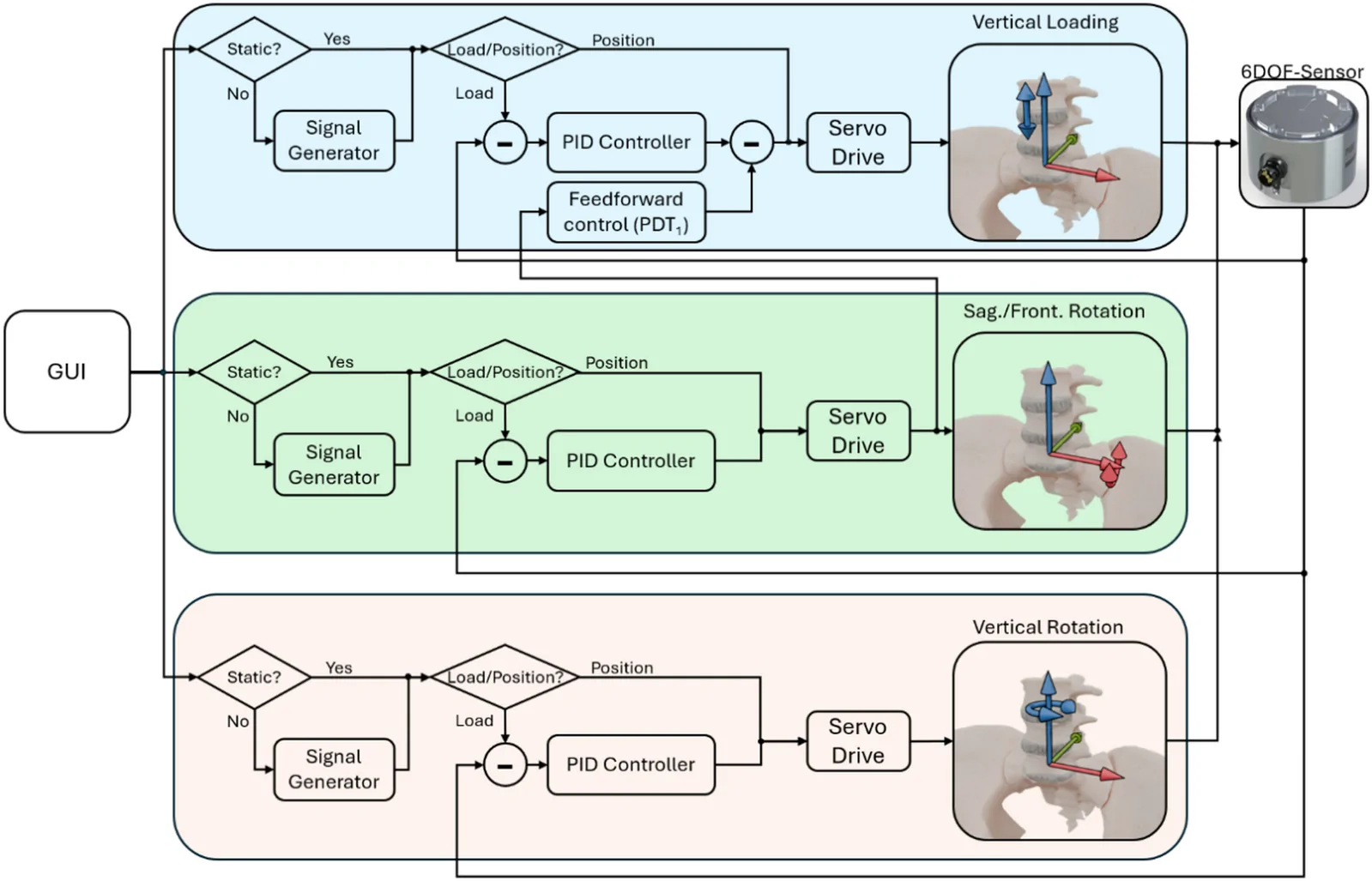
Schematic diagram of how the simulator works. Each coloured box shows the functionalities that can be set via the graphical user interface (GUI) and the corresponding control algorithm for one of the three directions of movement (blue: vertical loading; green: sagittal/frontal rotation; red: vertical rotation); The coloured arrows in the pictograms each represent a body axis (blue: vertical axis, red: frontal axis, green: sagittal axis); The measured values from the 6-DOF-sensor serve as feedback for the corresponding control loops.

The software is designed to allow each axis to be configured either to maintain a static load or position, or to perform a cyclic motion with a sinusoidal load or position. An overview of the control software architecture is shown in Fig. 2. When positions (linear positions or angles) are specified, these are passed directly to the integrated position control loops of the servo drives used. These integrated controllers use the measurement signal from the incremental encoders of the servo motors as a process variable. When static loads (forces or moments) or cyclic vertical loading are specified, they are transmitted as a setpoint to upper PID controllers. The PID controllers output positions as the control variable, which are passed on to the servo drive. The process variable for the PID controllers is the corresponding force or moment signal from the 6DOF sensor. When cyclic rotational loads are specified, they are transmitted to the integrated torque control of the corresponding servo drives. The torque control loops use the motor current as a process variable.

If the setpoint is specified statically, a constant setpoint is given to the respective controller. If the setpoint is specified cyclically, a setpoint generator is connected ahead of the respective controller, which outputs sinusoidal setpoint signals based on the previously set parameters. Thanks to the described structure of the control algorithm, the simulator is capable of implementing a wide range of motion and load profiles.

In addition to the PID controller described above, the control loop for the vertical crossbar has been extended to include a disturbance feed-forward mechanism in form of a PDT1 element. This is necessary because movements of the tilt platform to initiate extension or flexion raise or lower the specimen in question, and this constitutes a disturbance for the control loop of the vertical compression. When the platform moves quickly, a standard PID controller cannot compensate for these disturbances quickly enough, since it only reacts once a control error has already occurred as a result of the disturbance. The disturbance feedforward via PDT1 element, on the other hand, reacts directly to movements of the tilt platform and is connected to the output of the PID controller. With this extended control loop, the specified vertical load can be precisely achieved even during rapid extension or flexion. All PID controllers were set up according to the method of Chien, Hrones and Reswick and manually optimized^20^.

As the simulator has been designed for a wide range of specimens – from individual spinal motion segments to the pelvis with the adjacent spine extending into the thoracic region – each with very different geometric and biomechanical properties, the PDT1 element for disturbance feedforward must be adapted to the specific situation. During the development process, the authors found that, for this purpose, only the proportional component needs to be varied. Therefore, before moving or loading a new specimen, an automated tuning process is performed in which the tilting platform is slowly moved back and forward, and the PID control loop for vertical compression keeps the desired load at a constant level. The positions of the vertical axis reached during this process are set in relation to the positions of the tilting platform, and the P component of the PDT1 element is determined from this.

The control algorithm described, all the controllers it comprises, and the data acquisition system operate at a frequency of 1 kHz.

### Biomechanical testing

To validate the functionality of the developed simulator, two different test protocols were evaluated. Firstly, a range of motion analysis was carried out using the pure moments method, which is frequently employed in biomechanical studies of the spine^21–23^. In their review, Borrelli et al. stated that 60% of the studies they analyzed used this method to examine ROM. The method was described by Wilke et al. in 1998^18^. In this test, the spine is rotated in either torsion, flexion/extension or lateral bending at a speed of 1.5°/s until a moment of 7.5 Nm is reached in the respective load direction. All other degrees of freedom are kept unloaded during this process.

Secondly, cyclic flexion-extension loading of 5 Nm was applied under a constant axial preload of 60% body weight (BW) at 1 Hz. The preload was kept constant in the vertical direction, regardless of the angle of flexion, and is briefly based on Ruff’s work on weight distribution^24^. He described an average proportion of BW between 50% and 60% BW for the lumbar spine^11,24^. The results from Ruff are also available in the review by Costi et al.^11^. The moment for loading in the flexion/extension direction was derived from the findings by Costi et al. on spinal testing methods^11^. In their surveys, a majority of respondents stated that bending moments between 2.6 Nm and 7.5 Nm correspond to physiological loading^11^. As Schleifenbaum et al. found in their study that a bending moment of 7.5 Nm at 500 N of axial compression leads to frequent failure or loosening of pedicle screws^25^, the worst-case scenario was not selected; instead, a mid-range value of 5 Nm was used.

### Specimen preparation and mounting

The examinations described below were performed on five fresh frozen human donor specimens provided by the Institute of Anatomy at Leipzig University. The data for the corresponding body donors are listed in Table 1. All body donors gave their informed and written consent to the donation of their bodies for teaching and research purposes while alive. Being part of the body donor program regulated by the Saxonian Death and Funeral Act of 1994 (third section, paragraph 18, item 8), institutional approval for the use of the post-mortem tissues of human body donors was obtained from the Institute of Anatomy (University of Leipzig) by the Ethics Committee of the University of Leipzig Medical Center (ethical approval no. 129/21-ck, approval date March 1, 2023). The authors declare that all experiments were conducted according to the principles of the Declaration of Helsinki (as revised in 2013).

**Table 1:** Body donor characteristics of the specimens used.

Specimen	Age	Sex	Weight
1	86	female	42
2	90	male	85
3	80	female	75
4	84	female	66
5	95	male	70

Freshly frozen human specimens, which had been stored at −80 °C, were thawed overnight in a cold water bath prior to dissection and then prepared for instrumentation. For this purpose, the specimens were first severed between the L1 and L2 vertebral bodies, and the femoral heads were removed from the acetabula. The posterior surfaces of the lumbar vertebrae from L2 to the sacrum at the level of S1 were then cleared of muscle and fatty tissue near the entry points of the pedicle screws, exposing the periosteum. This ensured that the guides could be positioned precisely. Care was taken to leave the ligamentous apparatus intact so as not to compromise the stability of the spine. In addition, a partial osteotomy of the spinous processes and facet joints was required to ensure that the guides fit correctly. Subsequently, pedicle screws were placed bilaterally in the lumbar vertebrae from L2 to the sacrum at the S1 level. In addition, S2 alar-iliac (S2AI) screws were inserted bilaterally into the pelvis. Screw diameter, length and positioning were planned by an experienced surgeon using the MySpine surgical planning tool (Medacta International, Switzerland). In each case, the screw trajectory was planned to ensure the maximum length and diameter of the screw. Based on the planning, patient-specific templates (MySpine MCR, Medacta International, Switzerland) were fabricated and used for screw placement to ensure the previously planned position. With the templates in place, pilot holes were drilled using a 2.7 mm drill bit, a guide wire was inserted into the drill channels, and the templates were removed. The screw channels were then prepared using cannulated tap cutters. Finally, the pre-planned screws (M.U.S.T., Medacta International, Switzerland, Ti6Al4V and CoCrMo alloys) were inserted into the specimen and individually bent titanium rods (Ti6Al4V) were used to connect the screws longitudinally. ROM testing was performed without connecting rods, whereas cyclic loading testing was performed after rod implantation.

**Fig. 3:**
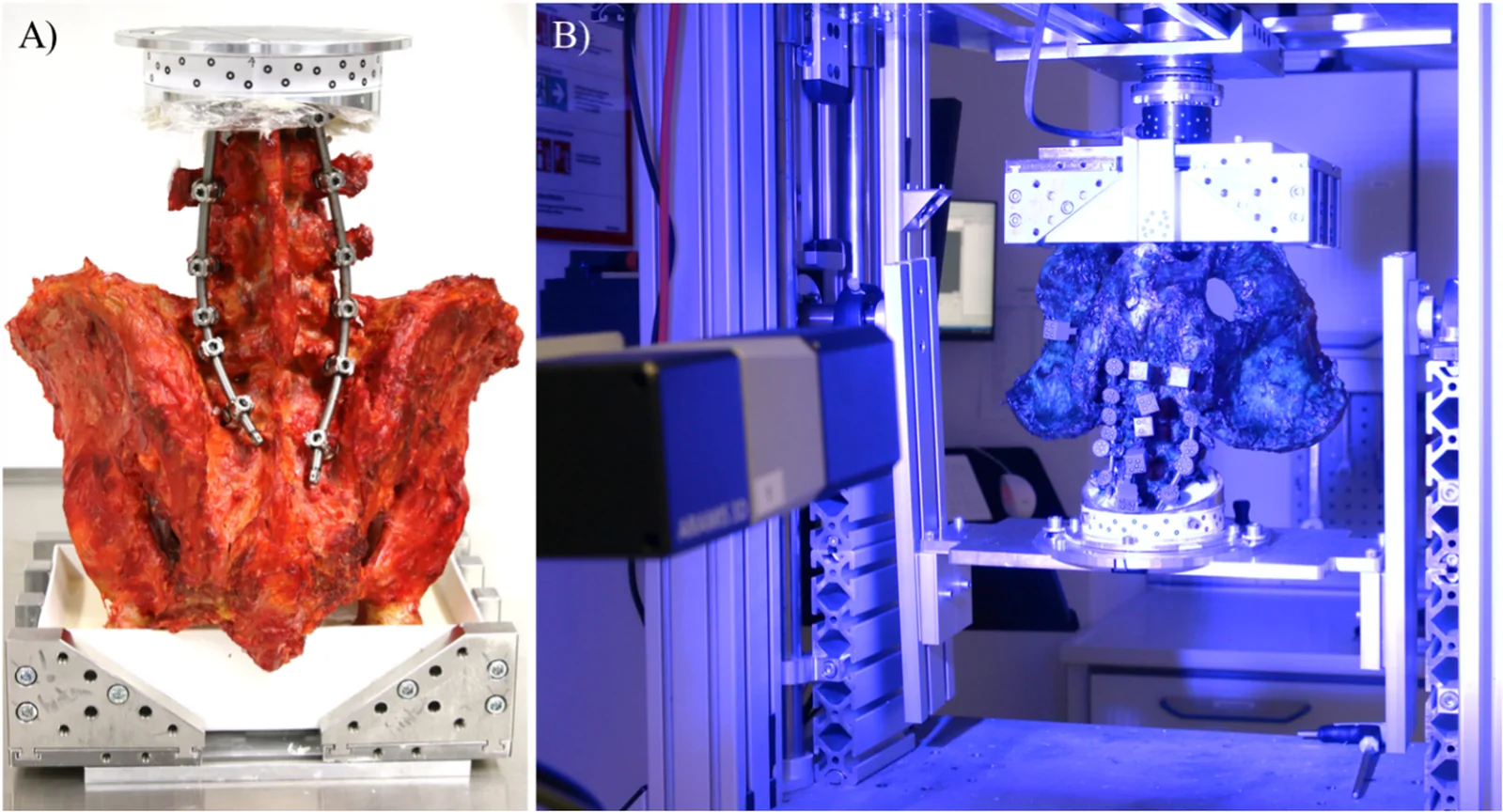
Test setup; (**A**): Cranial and caudal embedded specimen with implanted pedicle screw-rod-system; (**B**) Specimen with optical markers mounted in the simulator.

To secure the specimens in the test rig, both cranial and caudal bone structures were cast in PUR rapid casting resin (GP 010, Gößl + Pfaff GmbH, Brautlach, Germany). Prior to this, the bone structures were dehydrated using acetone for at least 15 min to enhance the adhesion of the resin to the bone surface and thus ensure a sufficient form-fit. Cranially, the L2 vertebra was fixed in a dedicated aluminium sleeve with a diameter of 120 mm (see Fig. 3A). The cured resin was screwed to the sleeve via four cross-shaped holes on the outer surface. Caudally, both ischial tuberosities were fixed in a 3D-printed container made of polylactic acid (PLA). This container was in turn clamped into a steel holder and screwed into the cured rapid casting resin via four holes (see Fig. 3A). During casting, the caudal container and the cranial sleeve were aligned parallel to one another using a protractor. Furthermore, both parts were positioned so that their centers were superimposed perpendicular to the base surfaces of the sleeve and the container. Before the specimen was secured in the simulator, the 6DOF sensor, together with the attached mounting plate and the steel holder, was zeroed to consider their influence on the load applied to the specimen. The sensor therefore indicated an unloaded state once the specimen’s own weight had been compensated for. Both the cranial and caudal fixations were connected to the test rig using four screws each (see Fig. 3B). The design of the simulator requires that the moment in the extension/flexion direction be applied from below. To ensure that the load is applied from a physiological direction, the specimen was mounted with the cranial end pointing downwards. This procedure was recommended by Schleifenbaum et al.^25^.

### Testing and data analysis

The prepared specimens were moved and loaded in the simulator using the two testing scenarios described. During this process, sensor data for all six degrees of freedom, as well as the position, and velocity of all three axes, were recorded at a frequency of 100 Hz and saved in a CSV file.

Each of the five specimens was first subjected to the ROM testing. The specimens were preconditioned for five cycles and afterwards five measurement cycles were recorded. The specimens were subsequently tested for 105 cycles using the cyclic load protocol described. The data were evaluated after 10 and 100 cycles, each over five cycles.

To quantify the agreement between the target and actual values (see Table 2), the mean absolute deviation (MAD) and the root mean square error (RMSE) between the target and actual values were calculated. The standard deviation (SD) of the difference from the target values was used as a measure of the precision of the actual values. The data was calculated using MATLAB (MathWorks, Natick, Massachusetts, USA).

During the ROM analysis, the data was first categorized by direction of movement (extension and flexion, left and right torsion, left and right bending) and then normalized to 100%. This means that a range from 0 to 100%, for example in the case of extension, describes the range between 7.5 Nm and − 7.5 Nm. For cyclic loading, a load cycle lasting one second is also normalized to 100%. In both cases, the respective five recorded cycles were averaged.

In addition to the MAD and RMSE for the entire cycles, six sub-ranges were defined for the cyclic loading, within which MAD and RMSE were calculated. One range was defined before (10–20%; 60–70%), around (20–30%; 70–80%) and after (30–40%; 80–90%) each of the two extrema.

**Table 2:** Target values of the two test scenarios used; For the actual value the source of the measuring values is given; Abbreviation 6DOF means the 6DOF sensor.

ROM	Extension-Flexion		Torsion		Bending	
	Target	Actual	Target	Actual	Target	Actual
F_Medio−Lateral_ in N	0.0	F (6DOF)	0.0	F (6DOF)	0.0	F (6DOF)
F_Anterior−Posterior_ in N	0.0	F (6DOF)	0.0	F (6DOF)	0.0	F (6DOF)
F_Superior−Inferior_ in N	0.0	F (6DOF)	0.0	F (6DOF)	0.0	F (6DOF)
M_Flexion−Extension_ in Nm	-	-	0.0	M (6DOF)	0.0	M (6DOF)
M_Lateral Bending_ in Nm	0.0	M (6DOF)	0.0	M (6DOF)	-	-
M_Axial Rotation_ in Nm	0.0	M (6DOF)	-	-	0.0	M (6DOF)
V_Rot_ in °/s	1.5	Encoder Servo	1.5	Encoder Servo	1.5	Encoder Servo
M_Rot, Max_ in Nm*	7.5	M (6DOF)	7.5	M (6DOF)	7.5	M (6DOF)

**Cyclic Loading**	**Extension-Flexion**					
	**Target**			**Actual**		
F_Superior−Inferior_ in N	60% BW			F (6DOF)		
M_Flexion−Extension_ in Nm	Sine (5 Nm, 1 Hz)			M (6DOF)		

## Results

**Specimens**

The experiments were carried out on five specimens as described. No fracture or other failures occurred during the experiments. The average age of the body donors was 87 ± 5.7 years and the mean body weight was 67.6 kg ± 16.0 kg. The specimens were derived from three female and two male body donors.

### ROM

The measurement values recorded by the simulator, covering the five unloaded degrees of freedom, as well as the angular velocity in the direction of loading are shown below as examples for the ROM analysis of the extension movement. The values were averaged across all five specimens. The mean force values ranged from − 0.15 N to −2.22 N in the medio-lateral direction, from 1.98 N to −3.15 N in the anterior-posterior direction, and from 0.32 N to −0.23 N in the superior-inferior axis direction.

**Fig. 4:**
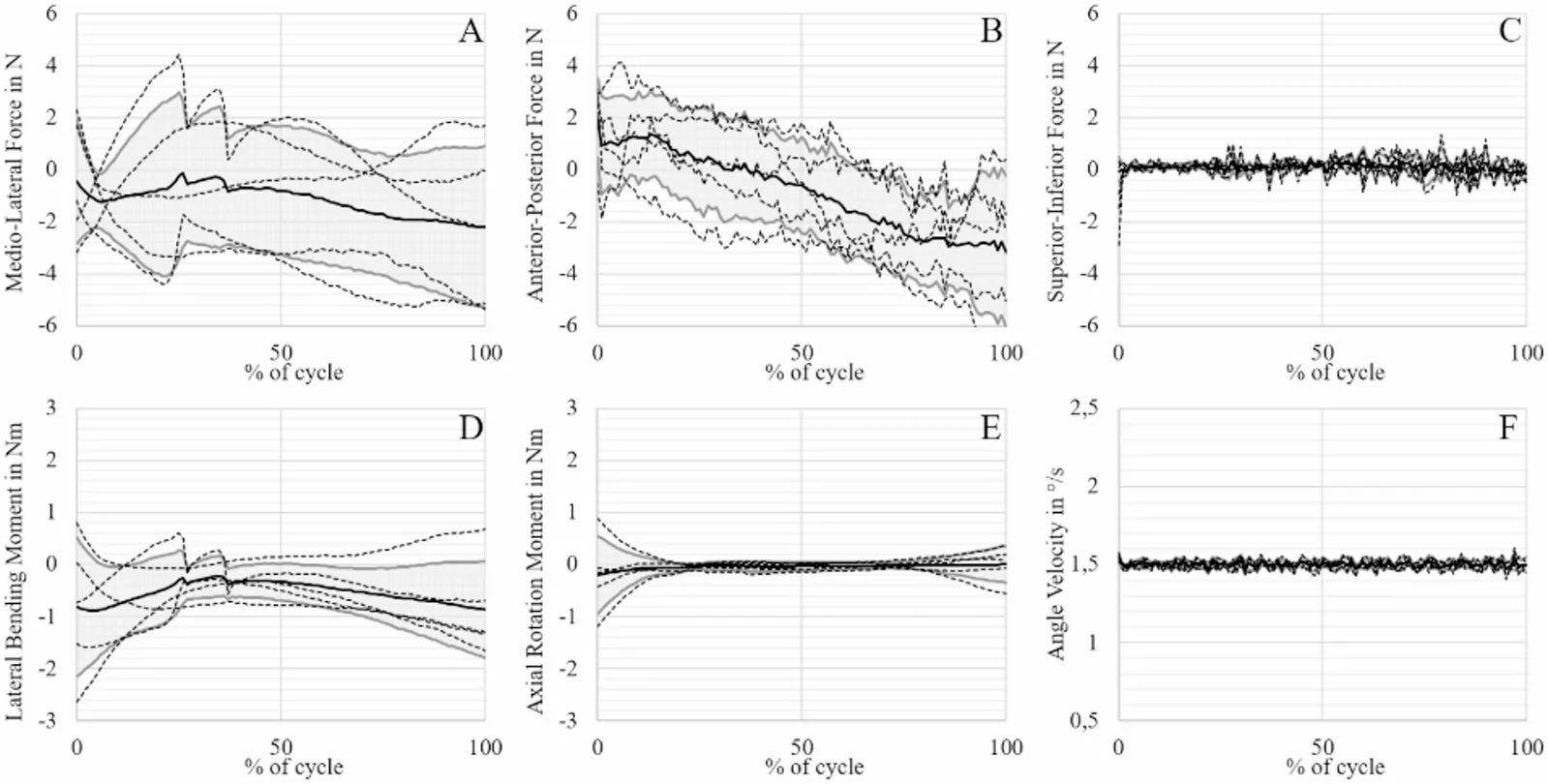
Measuring values of the simulator during ROM examinations using the example of extension movement. The figures show the mean curves across all cycles and all specimens (thick line) with standard deviation indicated and curves of each specimen (dotted lines) of (**A**) Medio-Lateral Force; (**B**) Anterior-Posterior Force; (**C**) Superior-Inferior Force; (**D**) Lateral Bending Moment; (**E**) Axial Rotation Moment; and (**F**) angular velocity – V_rot_.

The comparison between the recorded measurements and the specified load protocol during the ROM examination demonstrated good accuracy for all six movements. This comparison was quantified by the MAD, and the RMSE between the specified target value and the measured actual value (see Table 3). Except the anterior-posterior force during flexion and left and right bending, all MAD and RMSE values for the forces were below 5 N, which can be considered a good result. The results of all the movements carried out show the same behaviour based on these two parameters as in the extension shown as an example in Fig. 4. For all movements performed, the force in the superior-inferior direction deviates (MAD < 0.5 N; RMSE < 0.5 N) less from the target value than the forces in medio-lateral (MAD = 1.95 N.3.92 N; RMSE = 2.31 N.4.38 N) or anterior-posterior (MAD = 1.92 N.7.50 N; RMSE = 2.40 N.7.92 N) direction. Furthermore, all MAD and RMSE values for the moments were below 1 Nm. For extension-flexion and lateral bending, the angular velocities consistently show values of ≤ 0.05 °/s for both parameters. For torsion, both parameters show higher deviations of ≤ 0.23 °/s (MAD) and ≤ 0.31 °/s (RMSE).

**Table 3:** Accuracy results achieved when performing ROM examinations in different directions of movement, determined using the mean absolute deviation (MAD) and root mean square error (RMSE) compared to the target values (F = 0 N, M = 0 Nm, V = 1.5 °/s, M_max_ = 7.5 Nm).

Motion		Extension-Flexion		Torsion		Bending	
		Ext	Flex	Left	Right	Left	Right
MAD	F_Medio−Lateral_ in N	2.11	1.95	2.52	2.20	3.92	3.67
	F_Anterior−Posterior_ in N	1.92	7.50	4.21	3.68	5.27	5.07
	F_Superior−Inferior_ in N	0.25	0.30	0.12	0.12	0.24	0.27
	M_Flexion−Extension_ in Nm	-	-	0.60	0.46	0.74	0.69
	M_Lateral Bending_ in Nm	0.66	0.58	0.51	0.86	-	-
	M_Axial Rotation_ in Nm	0.12	0.13	-	-	0.22	0.23
	V_Rot_ in °/s	0.02	0.02	0.23	0.21	0.03	0.02
	M_Rot, Max_ in Nm*	0.10	0.01	0.09	0.01	0.18	0.01
RMSE	F_Medio−Lateral_ in N	2.59	2.31	3.09	2.60	4.38	4.11
	F_Anterior−Posterior_ in N	2.40	7.92	4.86	4.37	6.18	5.91
	F_Superior−Inferior_ in N	0.36	0.49	0.20	0.19	0.39	0.46
	M_Flexion−Extension_ in Nm	-	-	0.75	0.61	0.90	0.86
	M_Lateral Bending_ in Nm	0.80	0.73	0.64	0.99	-	-
	M_Axial Rotation_ in Nm	0.19	0.21	-	-	0.32	0.37
	V_Rot_ in °/s	0.03	0.03	0.30	0.26	0.03	0.03
	M_Rot, Max_ in Nm*	0.10	0.02	0.09	0.01	0.21	0.01

Although the individual specimens differed widely in terms of geometry, the measured values demonstrated good reproducibility. Table 4 shows the maximum and average standard deviation of the measured values as a measure of reproducibility. Measuring values were recorded for five samples. For all movements performed in the three force directions, the average SD was ≤ 3.56 N, and the maximum SD was ≤ 5.48 N. The mean SDs of the moments were ≤ 0.74 Nm. The maximum SDs of the moments, however, were considerably larger at ≤ 1.32 Nm; as can be seen in Fig. 4 (D and E), due to greater variations in the moments at the beginning and end of the motion cycles. The mean and maximum SD of the rotational speeds in the load direction showed excellent reproducibility with values of ≤ 0.11 °/s. Only the maximum SD for torsion was higher, at values of ≤ 0.27 °/s. The SD for the maximum moment also showed excellent reproducibility with values of ≤ 0.13 Nm.

**Table 4:** Reproducibility results obtained when performing ROM examinations in different directions of movement, determined using the maximum and mean standard deviation (SD) between the five specimens tested.

Motion		Extension-Flexion		Torsion		Bending	
		Ext	Flex	Left	Right	Left	Right
Maximum SD	F_Medio−Lateral_ in N	3.36	2.79	3.84	3.24	2.25	1.95
	F_Anterior−Posterior_ in N	2.91	3.14	2.91	2.44	4.97	6.00
	F_Superior−Inferior_ in N	1.30	1.00	0.63	0.60	1.69	1.20
	M_Flexion−Extension_ in Nm	-	-	0.91	0.87	1.27	1.20
	M_Lateral Bending_ in Nm	1.34	1.44	1.10	1.11	-	-
	M_Axial Rotation_ in Nm	0.75	0.85	-	-	0.66	0.75
	V_Rot_ in °/s	0.06	0.05	0.26	0.24	0.05	0.07
Mean SD	F_Medio−Lateral_ in N	2.45	2.22	1.91	1.51	1.78	1.46
	F_Anterior−Posterior_ in N	1.85	2.54	2.30	1.73	3.68	3.88
	F_Superior−Inferior_ in N	0.29	0.31	0.14	0.15	0.26	0.26
	M_Flexion−Extension_ in Nm	-	-	0.73	0.51	0.80	0.80
	M_Lateral Bending_ in Nm	0.57	0.45	0.54	0.52	-	-
	M_Axial Rotation_ in Nm	0.15	0.16	-	-	0.28	0.25
	V_Rot_ in °/s	0.03	0.03	0.10	0.09	0.03	0.03
SD	M_Rot, Max_ in Nm	0.04	0.01	0.03	0.00	0.11	0.01

### Cyclic loading

**Fig. 5:**
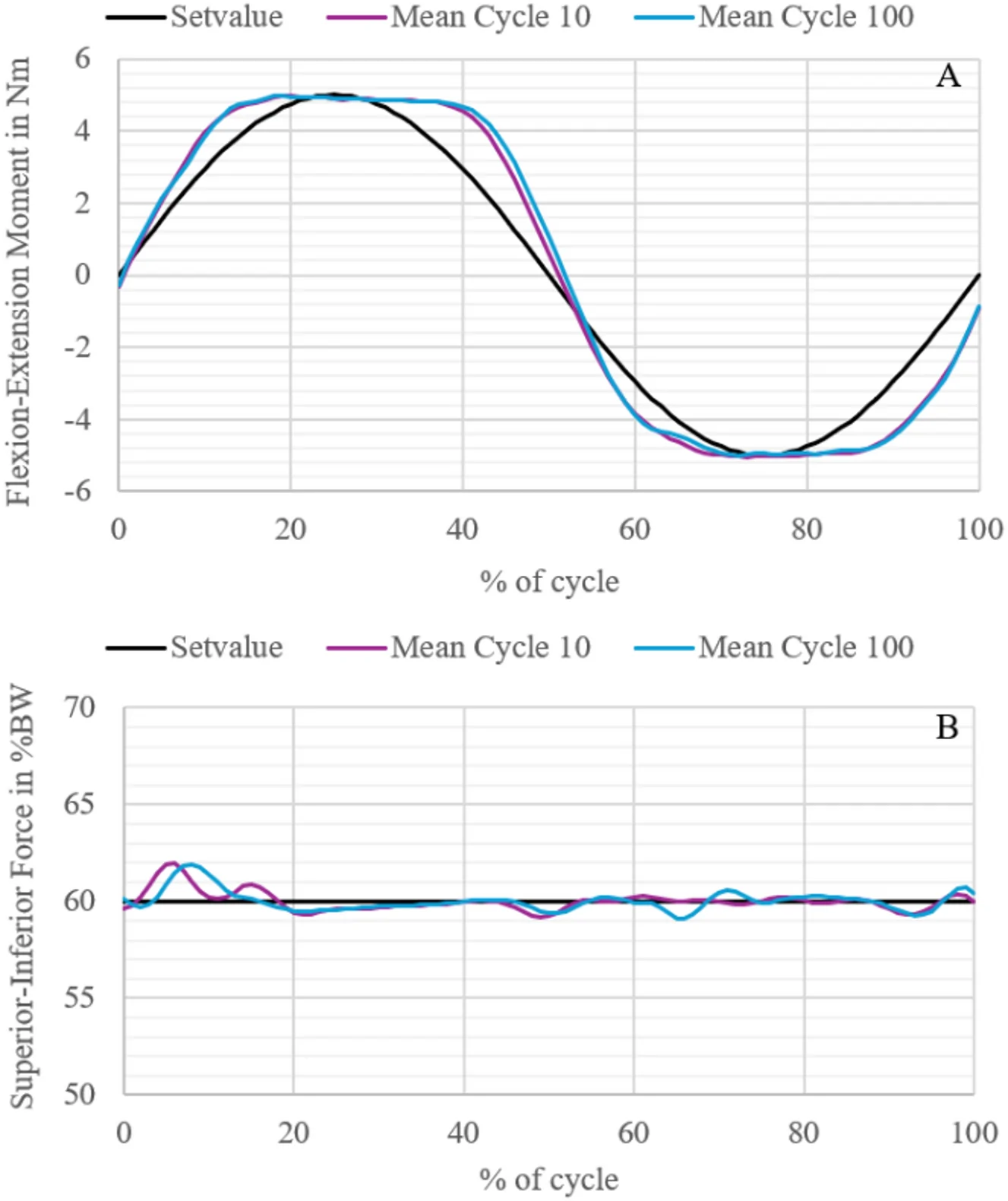
Measuring values of the simulator during cyclic loading compared to the given target value; (**A**) Flexion-Extension Moment; (**B**) Superior–Inferior Force.

To assess the accuracy and reproducibility of the simulator under cyclic loading, the results for the flexion-extension moment and superior-inferior force specified in the load protocol have been analyzed. Figure 5 shows the averaged measured values after 10 and 100 cycles compared to the specified target value. The amplitudes of the specified sinusoidal moment were adjusted very precisely along the curve of flexion-extension moment, which was also confirmed by the very small MAD and RMSE of ≤ 0.29 Nm (see Table 5). Furthermore, it was found that the deviations before and after the extreme values were three to five times greater (see Table 6).

The diagram shown at the bottom of Fig. 5 displays the averaged curve of the superior-inferior force in %BW of the respective body donors. The deviations determined as MAD and RMSE, were under 1.14%BW. The reproducibility of the superior-inferior force can be specified with a mean and maximum standard deviation of less than 2.10% BW.

**Table 5:** Accuracy and reproducibility results of the simulator while performing cyclic loading. *SD of M_Flex-Ext, Max_ and M_Flex-Ext, Min_ were calculated only once in each case.

Cycle	F_Superior−Inferior_ in *N*		F_Superior−Inferior_ in %BW		M_Flex−Ext, Sine_		M_Flex−Ext, Max_		M_Flex−Ext, Min_	
	10	100	10	100	10	100	10	100	10	100
MAD	2.87	2.94	0.73	0.76	0.72	0.77	0.09	0.10	0.23	0.15
RMSE	4.25	4.47	1.04	1.14	0.91	1.00	0.11	0.12	0.29	0.22
Maximum SD	7.31	7.72	1.69	2.10	0.56	0.83	0.11*	0.10*	0.19*	0.17*
Mean SD	3.41	3.43	0.83	0.89	0.26	0.30				

**Table 6:** Accuracy results of the simulator while performing cyclic loading, separated in sub-ranges before (10–20%; 60–70%), around (20–30%; 70–80%) and after (30–40%, 80–90%) the two extrema.

Cycle	M_Flex−Ext,10−20_		M_Flex−Ext,20−30_		M_Flex−Ext,30−40_		M_Flex−Ext,60−70_		M_Flex−Ext,70−80_		M_Flex−Ext,80−90_	
	10	100	10	100	10	100	10	100	10	100	10	100
MAD	0.67	0.73	0.13	0.11	0.83	0.85	0.59	0.54	0.17	0.16	0.85	0.85
RMSE	0.76	0.87	0.17	0.15	0.98	1.01	0.68	0.71	0.22	0.21	0.96	0.97

## Discussion

The aim of this study was to develop a versatile biomechanical loading simulator for the investigation of the pelvis, the spine and the lumbosacral junction, and to validate its functionality. This simulator is intended to enable biomechanical studies to be carried out on human specimens of these anatomical regions. To replicate a well-established test scenario, ROM testing was conducted using the pure moments method. A second test scenario was designed to demonstrate the functionality of the simulator in a typical clinical setting involving the lumbosacral junction and the pelvis. The results show that the developed simulator is capable of performing both established test protocols for determining range of motion using the pure moments method and multiaxial cyclic loading scenarios with high accuracy and reproducibility.

A key finding of this study is the generally good agreement between specified target values and measured actual values. This applies to both ROM investigations and cyclic loading. In particular, the low MAD and the low RMSE values for moments and forces demonstrate the high control quality of the system. The simulator thus fulfils a key requirement for biomechanical test benches, namely the reliable and reproducible representation of defined load and motion conditions. The results indicate that the simulator can reproduce established loading protocols with comparable accuracy and reproducibility^14^, supporting its suitability for preclinical biomechanical investigations.

Numerous studies have already examined the influencing factors and sources of error in biomechanical analysis of the spine^13,18,19,26^. Consequently, it is generally recommended to avoid shear forces when applying pure moments^18^ and/or axial loads or follower loads^11^. This requirement was met in the experimental setup presented here: in the transverse plane via a low-friction XY stage, as recommended by Gedet et al.^19^ and Ansapouri et al.^13^, and in the vertical axis via an actively driven and controlled crossbeam. The setup used, which allows for unrestricted translation, can lead to non-physiological translations when pure moments are applied^11^. To minimize this risk, care was taken during specimen preparation to leave all ligaments and facet joints intact, as these structures prevent non-physiological movements.

During the cyclic loading tests, it was demonstrated that the amplitudes of the applied moments were maintained with a high degree of accuracy and precision. At the same time, however, a lower degree of conformity with the sinusoidal load curve was observed, particularly in the regions around the extreme values. One plausible explanation for this is that the torque control of the servo drive was used to apply the load. In this process, the servo drive regulates the motor current, ensuring that the specified torque is applied to the output shaft. Due to dynamic effects of the system, such as the inertia of the specimen (including its embedding and the tilting platform), the deformation of spine and implant, and the load- and speed-dependent friction of the gearbox, the waveform of the moment at the servo drive’s output shaft may differ from that of the moment recorded by the sensor. The influence of these dynamic effects depends on the test frequency^15^. In the further development of the simulator, therefore, the position control of the servo drive is used to adjust the position of the tilting platform to minimize the tracking error. The current deviation from the sinusoidal moment signal should be considered a limitation of the simulator.

Unfortunately, the findings on accuracy and reproducibility when applying different loading protocols, as reported in this and other biomechanical studies, are difficult to compare directly. Due to the wide variety of test scenarios, for example in terms of the direction of the applied loads (vertical loads, perpendicular between the most cranial and most caudal vertebrae, or the so-called follower load)^12^, the control strategy used (force-controlled or position-controlled), or different output parameters, such as stiffness, there are generally only a few or no comparable studies. Furthermore, all studies conducted on human specimens are subject to individual differences in the specimens, which can vary greatly in terms of anatomy, bone quality or pathologies. To be able to compare different simulators in a standardized manner, the comparative tests would need to be conducted using standardized mechanical models, such as the one developed by Volkheimer et al.^27^.

Nevertheless, there are studies that allow for a comparison of the results presented here. For example, Kelly et al. also developed a simulator capable of simulating loads on the lumbar spine^14^. They use an actively driven and controlled servo drive for each of the six degrees of freedom. The validation of the simulator for the “pure moments” method was performed on similarly long segments of the spine (L1–S1); however, the angular velocity, ranging from 0.15 °/s to 0.5 °/s, was considerably lower than the 1.5 °/s used in this study. Since dynamics are a major factor influencing the tracking error^13,15,28^, the errors obtained by Kelly et al. are expected to be smaller. Based on the example of flexion-extension, it can be observed that the simulator presented here exhibits slightly smaller MAD of 0.25 N (extension) and 0.30 N (flexion) than Kelly et al. (0.49 N). In contrast, the simulator developed by Kelly et al. exhibits smaller MAD values for the medio-lateral forces (Kelly et al.: 0.52 N; this study: 1.95…2.11 N) and the anterior-posterior forces (Kelly et al.: 0.59 N; this study: 1.92…7.50 N). The MAD values obtained by Kelly et al. for the lateral bending moment (Kelly et al.: 0.02 Nm, this study: 0.58.0.66 Nm) and the axial rotational moment (Kelly et al.: 0.03 Nm, this study: 0.12.0.13 Nm) were also smaller.

To compare tracking errors under cyclic loading, the study by McGregor et al. can be consulted^28^. They used an AMTI joint simulator to apply cyclic moments (4 Nm) to a vertebral segment under constant axial compression (250 N). The maximum test frequency used here (0.3 Hz) was also lower than the 1 Hz frequency used in this study. Therefore, due to dynamic effects, smaller errors are also to be expected in this comparative study. McGregor et al. reported a mean RMSE of 16.3 N for the superior-inferior force during flexion-extension^28^. The errors presented here, 4.25 N (cycle 10) and 4.47 N (cycle 100), were considerably lower. Furthermore, their supplementary data includes a graph showing a RMSE in the flexion-extension moment of approximately 0.15 Nm^28^, which is substantially lower than the error of 0.91 Nm presented in this study.

In summary, the simulator is able to reproduce the specified superior-inferior force very well in comparison, shows less accurate agreement for dynamic flexion-extension moments, and can reproduce shear forces just as well as comparable simulators, given the higher dynamics used.

A key advantage of the simulator developed lies in its high flexibility in terms of installation space and the range of specimen geometries it can accommodate. Thanks to its adjustable structure, both small specimens, such as individual spinal motion segments, and large units, such as the pelvis with the adjacent spine extending into the thoracic region, can be examined. This versatility sets the test rig apart from many existing systems, which have often been developed for specific research questions or specimen geometries^15,16^. Furthermore, the simulator can apply extreme physiological compressive forces, such as those of 1166 N and above when holding a 20 kg weight against the body^11^. These compressive forces can be combined with bending moments of 10 Nm or greater, as applied in some studies^22,23,26,29^. Bending moments of up to 20 Nm can be applied, limited by the measuring range of the force sensor. Thanks to these capabilities, the simulator opens up a wide range of applications, from basic research to the evaluation of complex treatment concepts.

Despite the generally positive results, the study has several limitations. The validation was performed using only two load protocols, whereas additional multi-axis and highly dynamic loading scenarios remain to be investigated. Moreover, the influence of the mass and friction of the XT table has not yet been characterized, and no inertial decoupling has been implemented. Furthermore, no quantitative benchmarking against an established commercial or previously validated test system was possible. In addition, one rotational DOF is not considered in the design of the simulator, therefore the pure moments method can only be realized to a limited extent. In this study, this effect was minimized by aligning the cranial and caudal embeddings in parallel. This procedure is not suitable for the examination of samples with hyperlordotic curvature or spinal deformities. As a result, specimens with the aforementioned pathologies were not examined and cannot be examined given the current setup. It cannot be conclusively clarified whether the MAD of the lateral bending moment, which ranges from 0.51 Nm to 0.86 Nm and results from the missing rotational DOF, influence the ROM, as, to the authors’ knowledge, there is no study that explicitly examines the influence of a moment applied in addition to the pure moment. The sixth DOF will be considered in a subsequent revision of the simulator.

## Conclusion

In summary, the simulator developed demonstrates a high degree of accuracy, reproducibility and flexibility. It therefore provides a suitable platform for the biomechanical investigation of implants and stabilization concepts in the pelvic and spinal regions and offers a promising basis for future experimental studies.

## Data Availability

The datasets generated and analyzed during the current study are available from the corresponding author on reasonable request.
